# Melatonin: a potential intervention for hepatic steatosis

**DOI:** 10.1186/s12944-015-0081-7

**Published:** 2015-07-22

**Authors:** Hang Sun, Fang-fang Huang, Shen Qu

**Affiliations:** Department of Endocrinology and Metabolism, Shanghai Tenth People’s Hospital, School of Medicine, Tongji University, Shanghai, 200072 China

**Keywords:** Melatonin, Lipid metabolism, NAFLD

## Abstract

Melatonin (N-acetyl-5-methoxytryptamine, MLT) is a neuroendocrine hormone, which is primarily synthesized by the pineal gland in vertebrates. Melatonin is a remarkable molecule with diverse biological and physiological actions and is involved in the regulation of various important functions such as circadian rhythm, energy metabolism, the reproductive system, the cardiovascular system, and the neuropsychiatric system. It also plays a role in disease by having anti-neoplastic and anti-osteoarthritic effects among others. Recently, research has focused on the roles of melatonin in oxidative stress, lipid metabolism, and hepatic steatosis and its potential therapeutic roles.

## Introduction

**Fig. 1:**
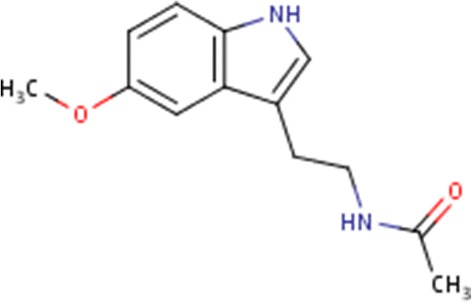
The molecular structure melatonin

Melatonin is a neuroendocrine hormone secreted by the pineal gland [1]. In mammals, the synthesis of melatonin is initiated by the transformation of tryptophan to 5-Hydroxytryptamine. 5-Hydroxytryptamine is converted into N-acetyl-5-hydroxytryptamine catalyzed by aralkylamine-N-acetyrltransferase (AANAT). After several additional reactions it is finally converted into N-acetyl-5-methoxytryptamine (Fig. 1) catalyzed by hydroxyindole O-methyltransferase (HIOMT) and this finally indole product is termed melatonin [2]. The synthesis and secretion of melatonin is regulated by light intensity [3]. Variations in light intensity stimulate the retinal ganglion cells to depolarize which stimulates the hypothalamic suprachiasmatic nucleus via the optic nerve. The hypothalamic suprachiasmatic nucleus releases adrenaline through the postsynaptic ganglion net, thus functioning in the pineal gland. Ultimately, the expression and activity of AANAT enzyme in pineal gland is up or down-regulated, and regulates the synthetic and secretion of melatonin.

Research has shown that [4] there is an obvious circadian rhythm in the blood levels of melatonin in vertebrates, with levels being higher at night and lower during the day. After synthesis, melatonin is released into the cerebrospinal fluid and blood immediately and then is distributed throughout the body in the systemic circulation.

### Biological function

Since it was discovered in 1958 [5] melatonin has become a hotspot for biomedical research. Initially, it was found that melatonin functions to regulate the sleep cycle, and melatonin production has been linked to the so-called ‘strong’ circadian oscillator [6].

Further investigations found that melatonin also has anti-tumor effects [7]. Melatonin can exert both direct and indirect anti-neoplastic effects in factorial synergy with other differentiating, anti-proliferative, immune-modulating and trophic molecules that form part of the anticancer treatment formulated by Luigi Di Bella [8]. The interaction between melatonin and the DBM (Di Bella Method, DBM: somatostatin, retinoids, ascorbic acid, vitamin D3, prolactin inhibitors, chondroitin-sulfate) molecules counters the multiple processes that characterize the neoplastic process (induction, promotion, progression and/or dissemination, tumoral genetic mutation).

Melatonin also functions in the regulation of immunity [9], resistance to ionizing radiation [10], and protection against free radicals and oxidative stress [11]. It also protects against ischemia-reperfusion injury after myocardial [12]. Further research has shown that it can regulate lipid and glucose metabolism [13–15].

Two types of melatonin receptors have been identified melatonin membrane receptor (MMR) and melatonin nuclear receptor (MNR). There are three types of melatonin membrane receptors which have been named MT1, MT2, MT3. Among them, both MTl and MT2 are G-protein-coupled receptors, which are widely distributed in the nervous, cardiovascular, immune, and reproductive systems. [16, 17] MT3, which is also called chinone reductase-2, belongs to the reductase family. It has been found in various tissues and organs in mammals [18]. MT3 can inhibit the generation of reactive oxygen species (ROS) in mitochondria by preventing the entry of reducing equivalents from chinone into the electron transport chain. The melatonin nuclear receptor isretinoid Z receptor alpha (RORa/RZR), but its function is still unclear. Moreover, melatonin has also been found to be a highly fat soluble hormone, which can enter cells independently of membrane receptors and bind with nuclear receptors or other transcription factors [19].

### Antioxidation function

Melatonin is a strong antioxidant and acts as a free radical scavenger [20]. It can scavenger superoxide (O^−^), hydrogen peroxide (H_2_0_2_), hydroxyl radicals (OH^.^), organic peroxy radicals (ROO^.^), singlet oxygen (^1^0_2_), nitric oxide (NO^.^), peroxynitrite (ONOO^−^), and hypochlorous acid. Melatonin can activate antioxidant enzymes [21], and can regulate gene transcription of antioxidant enzymes [22]. In a study adriamycin provoked lipid peroxidation and subsequent induction of oxidative-stress related genes were down-regulated reflecting the reduced oxidative stress in livers of the melatonin treated animals [23]. Melatonin can stimulate the secretion and activity of glutathione synthase [24], glutathione peroxidase(GSH-Px), glutathione reductase(GSH-Rd), catalase(CAT), AND glucose-6-phosphate dehydrogenase [25, 26]. Melatonin also causes a significant reduction in oxidative stress by reducing plasma malondialdehyde (MDA) levels and an increase in plasma superoxide dismutase (SOD) levels [27]. Melatonin can down-regulate the expression of some enzymes which increase oxidative stress such as nitric oxide synthase (NOS) [28]. Melatonin treatment abrogated oxidative stress in the liver of aged rats by preventing of the decreased activity of CAT and the downregulation of Cu,Zn-SOD and GPx gene expression [29]. It can augment the activity of other antioxidants [30] and protect antioxidant enzymes from oxidative damage [31], Melatonin acts on the mitochondrial respiratory chain decreasing electron leak and lowers the generation of free radicals [32]. Melatonin can also increase the synthesis of ATP [33].

### Anti-inflammatory function

Many studies have found that melatonin has anti-inflammatory effects. Experiments using melatonin as a pharmaceutical agent were mainly *in vitro* studies where melatonin doses exceeding the nocturnal plasma levels are required to exert clear effects. At supra-physiological concentrations, melatonin induces T-cell proliferation and up-regulation of pro-inflammatory cytokines [34, 35]. Hu et al. found that melatonin treatment significantly decreased the severity of hepatic cell damage, steatosis and the immigration of inflammatory cells, reduced serum and tissue inflammatory cytokines levels, tissue lipid peroxidation, neutrophil infiltration and inhibited the apoptosis of hepatocytes in an alcohol-induced hepatic injury mice model [36]. Exogenous melatonin administration increases the proliferative response of rat lymphocytes [37], increases the number of NK cells [38], stimulates the pro-inflammatory cytokines IL-1 and TNF-α [39, 40] and enhances phagocytosis [41]. Melatonin exerts a concentration-dependent effect on the immune system. Indeed, increasing concentrations of melatonin induce T-cell proliferation in a dose-dependent way. In addition, it was demonstrated that pharmacological doses of melatonin inhibit

INF-γ production at concentrations of 0.1–1 mM [42, 43]. In some systems, the modulation of apoptosis requires high melatonin doses [44, 45]. Whether or not these effects have physiological significance, occurring as a paracrine or autocrine response, i.e., in microenvironments with an elevated melatonin concentration like in the bone marrow, is currently being investigated. In any case, these findings demonstrate that melatonin is a potential exogenous pharmacological modulator of the inflammatory response.

### Effects on insulin resistance

Recent studies have confirmed the presence of the melatonin membrane receptors MT1 and MT2 in human pancreatic tissue and the islets of Langerhans [46]. The expression of melatonin receptors in patients with T2DM was demonstrated in immunohistochemical studies [47]. It was found that melatonin receptors on pancreatic β-cells are involved in three parallel signaling pathways, which have different effects on insulin secretion. In terms of insulin release, the insulin-inhibiting action of melatonin is mediated by the dominantly expressed MT1 receptor through activation of Gi-coupled adenylate cyclase activity, thereby negatively modulating incretin-induced increase in 3’, 5’-cyclic adenosine monophosphate (cAMP). Likewise, it was also demonstrated that melatonin inhibits the 3’, 5’-cyclicguanosine monophosphate (cGMP) signaling pathway and, consequently, insulin secretion possibly in a MT2 receptor mediated fashion. Meanwhile, melatonin-dependent IP3 release may play a role in the short-term support of other IP3-releasing agents, like acetylcholine, or may be related to the long-term regulation of pancreaticcell functions which may affect insulin secretion [15]. All of these studies support the concept that melatonin plays an important role in the regulation of insulin secretion and glucose/lipid metabolism.

### Effect of melatonin on lipid metabolism

Along with the increased understanding of the functions of melatonin, more research has focused on the effects of melatonin on lipid metabolism and hepatic steatosis.

Early animal experiments showed that high fat diet induced hyperlipidemia [48]. Melatonin treatment for three months can significantly reduce serum total cholesterol (TC) and low density lipoprotein (LDL-C) levels and remarkably increase high density lipoprotein (HDL-C) [49]. Long-term treatment with melatonin(1.1 mg/day for 30 weeks)can significantly reduce the liver lipid content in type 2 diabetic rats. Melatonin administration to Otsuka Long-Evans Tokushima Fatty(OLETF)rats reduced the hypertriglyceridemia by 39 % and hyperinsulinemia by 33 % and also restored hepatic delta-5 desaturase (an enzyme which aid in insulin secretion) activity by 148 %. Research on high-fat diet (HFD)-induced obesity in animal models has showed that [27] compared with an untreated obese group and a lean control group, melatonin treatment for four weeks causes a statistically significant decrease in serum lipids, an increase in GSH-PX and HDL, and reversal of fatty changes in the liver and atherosclerotic changes in the blood vessels. These studies suggest that exogenous melatonin administration has effects on lipid metabolism and provides the basis for additional research.

Subsequently, researches in human subjects also confirmed the beneficial effect of melatonin on lipid metabolism. Kadmin et al. reported that dyslipidemia was improved in patients with type 2 diabetes after treatment with melatonin [50]. This study was designed to evaluate the effects of melatonin and zinc on the lipid metabolism and renal function in type 2 DM patients poorly controlled with metformin. A placebo-controlled, double-blind clinical trial was performed in which 46 type 2 diabetic patients were selected and allocated into three groups. These groups were treated with single daily oral doses of both 10 mg melatonin and 50 mg zinc acetate or 10 mg of melatonin and 50 mg of zinc acetate in addition to the regularly used metformin or placebo over a period of 90 days. Fasting lipid profiles and microalbuminuria (MAU) were measured before initiating the treatments (zero time) and after 30 and 90 days of treatment. Daily administration of melatonin and zinc improved lipid metabolism and decreased levels of MAU as well as improving the response to metformin.

Kozirog M et al. found that melatonin treatment improves lipid metabolism in patients with metabolic syndrome [51]. Melatonin administered for 2 months (5 mg/day, 2 hr before bedtime) significantly improved lipid profile (decrease in low-density lipoprotein cholesterol 149.7 ± 26.4 versus 139.9 ± 30.2 mg/dL, *P* < 0.05) and also lowered blood pressure (systolic blood pressure - 132.8 ± 9.8 versus 120.5 ± 11.0 mmHg, *P* < 0.001, diastolic blood pressure - 81.7 ± 8.8 versus 75 ± 7.4 mmHg, *P* < 0.01).

### Effect of melatonin on liver fat accumulation

Research has shown that liver steatosis can lead to insulin resistance as well as dysfunction of glucose and lipid metabolism. Nonalcoholic fatty liver disease (NAFLD) is an early reversible accumulation which can lead to steatohepatitis and has recently been the subject of a considerable amount of research. Pan et al. [52] found that melatonin ameliorates NAFLD induced by a high-fat diet in rats. A high-fat diet leads to oxidative stress with extensive liver steatosis in rats. Melatonin reduced hepatic steatosis and inflammation by lowering serum AST, ALT, liver total cholesterol, and triglycerides in high-fat diet fed rats.

Further studies have suggested that melatonin can decrease hepatocyte apoptosis [53]. Methionine- and choline-deficient diet-induced nonalcoholic steatohepatitis rats were treated with melatonin (50 mg/kg/day, intraperitoneally) for one month. It was observed that melatonin decreased oxidative stress, levels of proinflammatory cytokines, and hepatocyte apoptosis.

A study on high-fat-diet rats by Gregorios Hatzis et al. [54] found that compared to untreated group, the two treated groups with different doses of melatonin (5 or 10 mg/kg) both had decreased mean liver weights (−5.0 g and −4.9 g) (*P* < 0.001) and lower weight ratios of liver weight to body (−1.0 %) (*P* < 0.001). Rats fed a high fat diet treated with melatonin showed significantly less steatosis. The group treated with 10 mg/kg melatonin exhibited grade III steatosis in 1 of 29 (3.4 %) rats, while the group treated with 5 mg/kg melatonin exhibited grade III steatosis in 3 of 27 (11.1 %) rats. However, melatonin could not reverse established steatosis.

Celinski et al. demonstrated the effect of melatonin on patients with NAFLD [55]. Essentiale with tryptophan (2 x 500 mg/day) or melatonin (2 x 5 mg/day) for 14 months both have a significant reduction in the activity of gamma-glutamyl transferase (GGPT) and levels of triglycerides and LDL-cholesterol comparing with the control group treated with Essentiale forte only. In the liver biopsies performed before and after treatment, melatonin and tryptophan reduced macrovesicular steatosis and lobular inflammation, while there was no change in control group.

### Possible mechanisms

While the true mechanism of action of melatonin on lipid accumulation remains under investigation, several theories have been suggested.

Lipid peroxidation and lipid membrane peroxidation contribute to the progression from steatosis to steatohepatitis, liver cell necrosis, necroinflammation, elevated transaminases, and fibrosis [56]. However, steatosis would not progress to steatohepatitis and fibrosis were it not for the contribution of free radicals and oxidative stress [57, 58]. Inhibition of the electron transport chain could lead to the generation of superoxide, which is capable of initiating lipid peroxidation and the generation of addition oxidative stress [59]. Melatonin can significantly reduce the activation of LPS-induced sterol regulatory element-binding protein (SREBP)-1c, as well as the expression of SREBP- 1c target genes which can prevent LPS-induced hepatic lipid accumulation [60]. In this respect, as a free radical scavenger, melatonin may have additive effects on the reduction of lipid peroxidation.

**Fig. 2:**
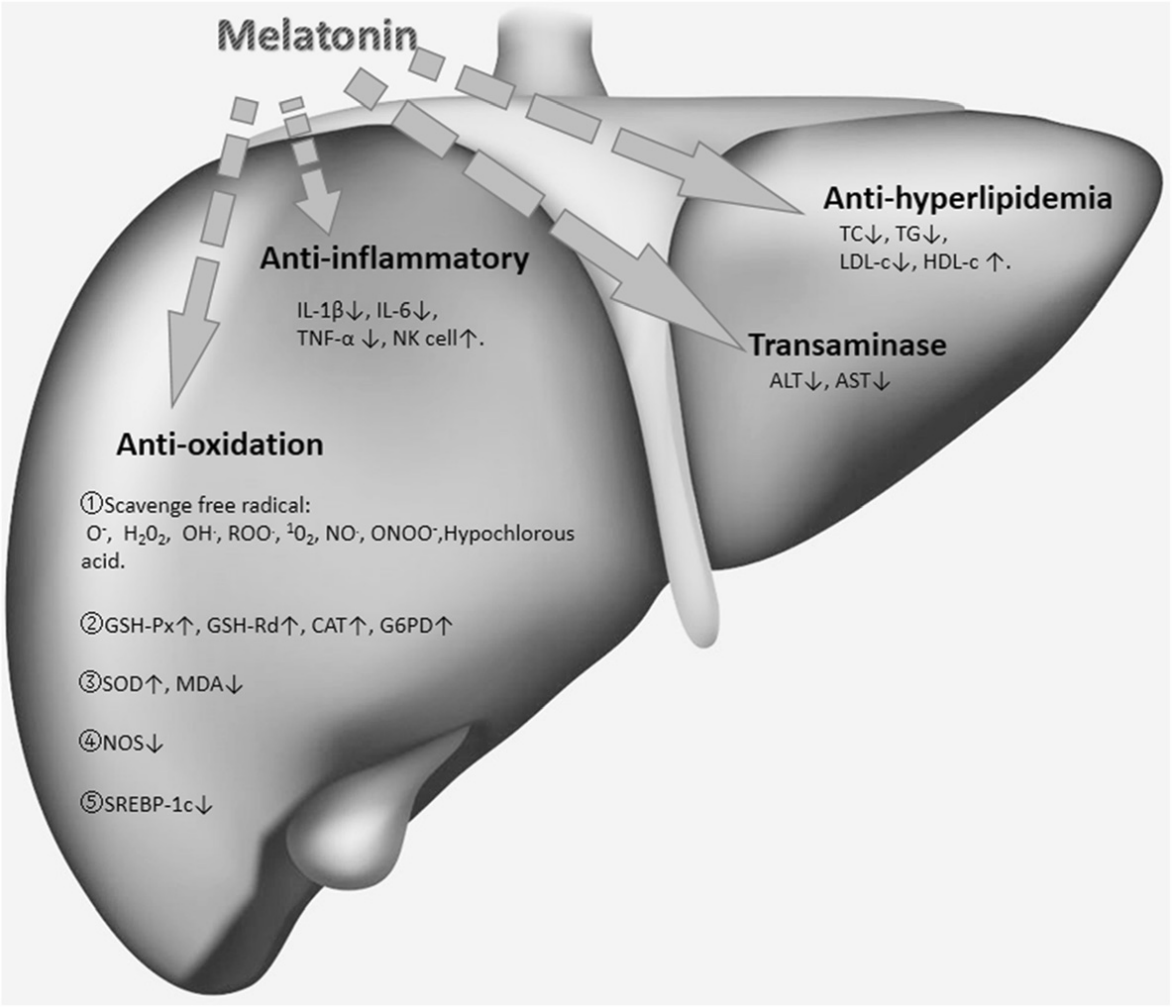
Possible mechanism of action of melatonin on improving dyslipidemia and hepatic steatosis. (O^−^ = superoxide; H_2_0_2_ = hydrogen peroxide; OH^.^ = hydroxyl radicals; ROO^.^ = organic peroxy radicals; ^1^0_2=_ singlet oxygen ; NO^.^ = nitric oxide; ONOO^−^ = nitric oxide; GSH-Px = glutathione peroxidase; GSH-Rd = glutathione reductase; CAT = catalase; G6PD = glucose-6-phosphate dehydrogenase; SOD = superoxide dismutase; MDA = malondialdehyde; NOS = nitric oxide synthase; SREBP-1c = sterol regulatory element-binding protein-1c; IL-1β = interleukin-1β; IL-6 = interleukin-6; TNF-α = Tumor Necrosis Factor-α; NK = Natural Killer; IL-1 = interleukin-1; ALT = Alanine transaminase; AST = Aspertate Aminotransferase; TC = total cholesterol; TG = Triglyceride; LDL-C = low density lipoprotein; HDL-C = high density lipoprotein.)

The mechanism of liver injury was found to be related to increased levels of pro-inflammatory cytokines, oxidative stress, and pro-apoptotic factors. Liver steatosis and apoptosis were found to occur in methionine-, choline- and folate-deficient diet rats, which is considered to be related to inflammation [61]. Melatonin can prevent liver damage by inducing the hepatic apoptotic index in these methionine-, choline- and folate-deficient diet rats model. Through increasing the migration of inflammatory cells and their activation, oxidative stress plays an important role in the development of liver inflammation. Methane Dicarboxylic Aldehyde (MDA), the final product of lipid peroxidation, has been shown to be closely related to the level of inflammation [62]. In a recent study by Kireev et al. [63], it was observed that melatonin can protect the liver of aged ovariectomized rats from pro-inflammatory and oxidative damage. Compared with untreated rats in the control group, both the two melatonin-treated groups of intact and ovariectomized rats demonstrated a significant reduction in the level of oxidative stress; decreased levels of IL-1β, IL-6, TNF-α, and also decreased transaminases, and tissue MDA. However, the melatonin treated groups demonstrated increased levels SOD and GSH. Melatonin’s efficacy on the biochemical and histopathological markers of the liver damage is probably associated with its anti-inflammatory effects (Fig. 2).

## Conclusion

In conclusion, melatonin has significant anti-oxidation and anti-inflammatory activities. In various studies in animals and human, melatonin has been shown to be beneficial not only in lipid metabolism, but also in improving liver fat accumulation and insulin resistance as well. As a regulator of various systems, melatonin may prove to be an important therapeutic choiceto improve dyslipidemia and hepatic steatosis.
